# Initial signs in patients with ruptured abdominal aortic aneurysms: time for an expanded triad?

**DOI:** 10.1186/s13049-024-01268-0

**Published:** 2024-09-23

**Authors:** Peter Bergmark, Mitra Sadeghi, Mareia Talvitie, Rebecka Hultgren

**Affiliations:** 1 Emergency Department, Karolinska University Hospital Stockholm, Stockholm, Sweden; 2 Department of Vascular Surgery, Karolinska University Hospital Stockholm, Stockholm, Sweden; 3 Stockholm Aneurysm Research Group, STAR, Department of Molecular Medicine and Surgery, Karolinska Institutet, Stockholm, Sweden

**Keywords:** Aortic aneurysm, Abdominal, Aneurysm, Ruptured, Symptom assessment, Missed diagnosis, Diagnostic errors, Mortality

## Abstract

**Background and objective:**

Misdiagnosis of ruptured abdominal aortic aneurysms (rAAA) contributes to delayed treatment and potentially higher mortality. The symptomatology in patients with rAAA is complex and challenging, 25–50% presumably fulfill the criteria of the standard triad of signs (STS). The objective was to determine the initial signs registered for patients with verified rAAAs, and to investigate if an expanded diagnostic triad could increase the diagnostic accuracy.

**Methods:**

A population-based study was conducted among all patients presenting with verified rAAAs in Stockholm County, Sweden, from January 2010 to October 2021. Patients were identified with ICD code 171.3 (rAAA). The STS was defined as (1) abdominal pain, (2) syncope and (3) the finding of a pulsatile abdominal mass, the prevalence of STS was investigated. An expanded triad included similar and related signs commonly registered for patients with rAAA, and was referred to as the *modified abdominal aortic aneurysm rupture signs* (MARS). The MARS-signs encompassed (1) the registered pain-associated symptoms or signs, (2) all hypovolemic associated signs, and (3) pulsatile abdominal mass and/or ultrasound finding, and the prevalence was similarly investigated. Finally, the STS and MARS were compared to evaluate the usefulness and performance of the MARS-score.

**Results:**

A total of 216 patients were identified. The majority were men (77%) with a median age of 78 years. The dominating symptom was abdominal pain (84%), followed by dizziness (50%). Few patients presented with three STS (13%), two STS were found in 37% and one STS in almost half of the patients (41%). By contrast, when applying MARS 35% presented with the complete expanded triad, 47% with two and 17% with one. Comparison of accuracy favored MARS (13 vs. 35% with 3 signs, *P* < 0.001 for STS vs. MARS) (2 or 3 signs, 48 vs. 82% STS vs. MARS, *P* < 0.001).

**Conclusions:**

The expanded MARS-signs could aid in easier and faster identification of rAAA patients, thus facilitating the first step with accurate diagnosis into the lifesaving rAAA care chain. Supportive diagnostic mnemonics and tools are especially important when targeting fatal diagnoses such as rAAA. Further studies are needed to investigate the implementation of the MARS-signs in various clinical settings.

**Supplementary Information:**

The online version contains supplementary material available at 10.1186/s13049-024-01268-0.

## Introduction

Patients with ruptured abdominal aortic aneurysm (rAAA) present with high pre- and intra-hospital mortality rates, persisting at 20–60% even with emergent surgical treatment [1–4].

One of the challenges is to identify and diagnose rupture in time [5–11]. Patients presenting with rAAA in the emergency department require rapid triaging due to the high morbidity and mortality occurring within the first hours [12].

Patients with suspected rAAA have for many decades been evaluated in the emergency department by means of a triad of clinical signs, the standards triad of signs (STS): (1) abdominal pain, (2) hypotension and (3) a pulsatile abdominal mass [13]. Due to atypical symptoms and impalpable aneurysm the signs of the triad is quite commonly absent, the STS underestimates the occurrence of rAAA up to 75% [14–16].

The diagnostic validity of the triad is uncertain and might partially explain the frequent misdiagnosis of rAAA in the emergency room. Misdiagnosis is known to be common, leading to higher rate of complications and mortality [17]. Studies have shown that 30–40% of patients with rAAA were initially misdiagnosed at the primary assessment in the emergency department, women more often than men [14, 17–19]. The most common incorrect differential diagnoses include kidney stones or myocardial infarction, resulting in a delay of important and potentially lifesaving treatment [14–16, 18]. Due to suboptimal performance, several other risk scores have been developed to aid clinicians and improve outcomes. Unfortunately, a sustained risk of misdiagnosis remains [19, 20]. Ruptured abdominal aortic aneurysm can present itself in a variety of ways, which contributes to the common misdiagnosis or delayed diagnosis. Studies of the varied panorama of symptoms in rAAA have been sparse; even fewer studies have been population-based. Further efforts and investigations into the symptomatology of rAAA are warranted to widen the understanding of its clinical presentation.

The primary interest of the clinical and scientific communities regarding rAAA patients has been in the post-diagnostic area including method of chosen intervention for patients with rAAA or survival rates after intervention [12, 21, 22]. This can be shown by the paucity of contemporary publications regarding diagnostic accuracy in rAAA patients. The primary aim of this study was to investigate the symptomatology documented for patients with verified rAAA. A specific objective was to assess the STS and explore if an expanded, modified triad could be helpful in the clinical setting, and increase the possibility of a rapid correct diagnosis.

## Material and method

### Study design

This is a population-based retrospective multicenter study on registry-based data from patients with diagnosed rAAA within Stockholm County, from 2010 to 2021.

The Stockholm County region had a population of 2.3 million inhabitants (2019). There are seven hospitals with emergency medical care units, of which two provide elective and emergent vascular surgery for the whole region in a combined on-call service (Karolinska University Hospital and Stockholm South General Hospital). All patients in the region with vascular emergencies are admitted or referred to one of these two centers, approximately 55–59% of all vascular patients will be admitted to the Karolinska University hospital. There is always a vascular surgeon on-call for vascular emergencies working at this hospital. Patients with or without verified rAAA deceased at other hospitals, that are not referred are not included.

### Study cohort and definitions

**Fig. 1:**
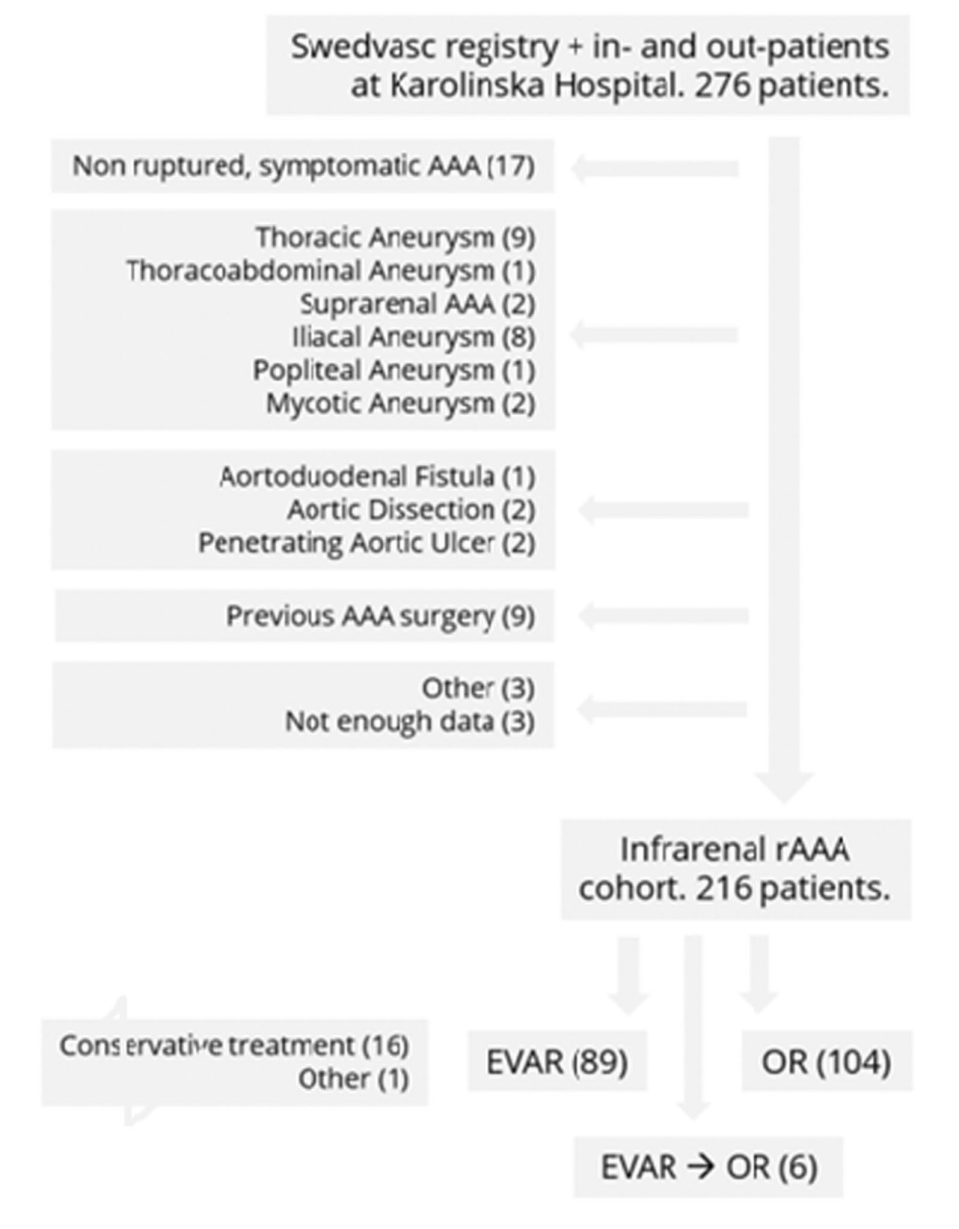
Flow chart of included patients with ICD code I71.3, based on the primary cohort of infrarenal rAAA cohort identified through the EMR, selection, exclusion, and inclusion criteria

The cohort was extracted by two means; from the Swedish National Registry for Vascular Procedures, Swedvasc [3], which registers all vascular interventions performed at the Karolinska University Hospital since 1994 with an external validity of (98–100%), and the in-hospital registry for diagnosed rAAA patients, including all rAAA patients admitted to the Karolinska University Hospital, thereby also catching all admitted but untreated patients with certified rAAA. Figure 1.

Patient Identity Number (PIN) and data from all patients with verified rAAA, untreated or treated with either open or an endovascular aortic repair were collected. A total of 277 patients were identified from January 2010 to October 2021 in the Karolinska University Hospital (Fig. 1). Patients diagnosed with rAAA not selected for vascular intervention were identified through the international classification of disease-10th edition (ICD-10), which uses ICD-code I71.3 for verified rAAA, at Karolinska University Hospital. A total of 276 patients were identified using Swedvasc registry and the Karolinska hospital electronic medical records (EMR).

This leaves one missing patient with a rAAA-diagnosis, lacking a clear description of care or treatment. Conservative treatment with palliation was chosen for 16 patients. After exclusion of non-infrarenal aortic ruptures, a total of 216 patients were identified and included (49 women and 167 men). In total, sixty patients were deemed not eligible for inclusion (Fig. 1).

Data was collected regarding comorbidities and risk factors for vascular disease as registered in any note in charts, any time prior to the event of rupture. Weight and height were commonly provided in the EMR. Radiological findings, i.e., computer tomography and, or ultrasound at admission were registered.

Clinical symptoms (abdominal, back, flank, groin and chest pain, dizziness, syncope, nausea, vomiting, dyspnea, pulsatile abdominal mass) were registered as stated in the EMR.

Dizziness was expanded in this study to include terms such as “about to faint”, “pale” and cold/shivering since it was commonly described and in general terms were evaluated as equivalents to dizziness in this study.

### Modified abdominal aortic aneurysm rupture signs, MARS

**Fig. 2:**
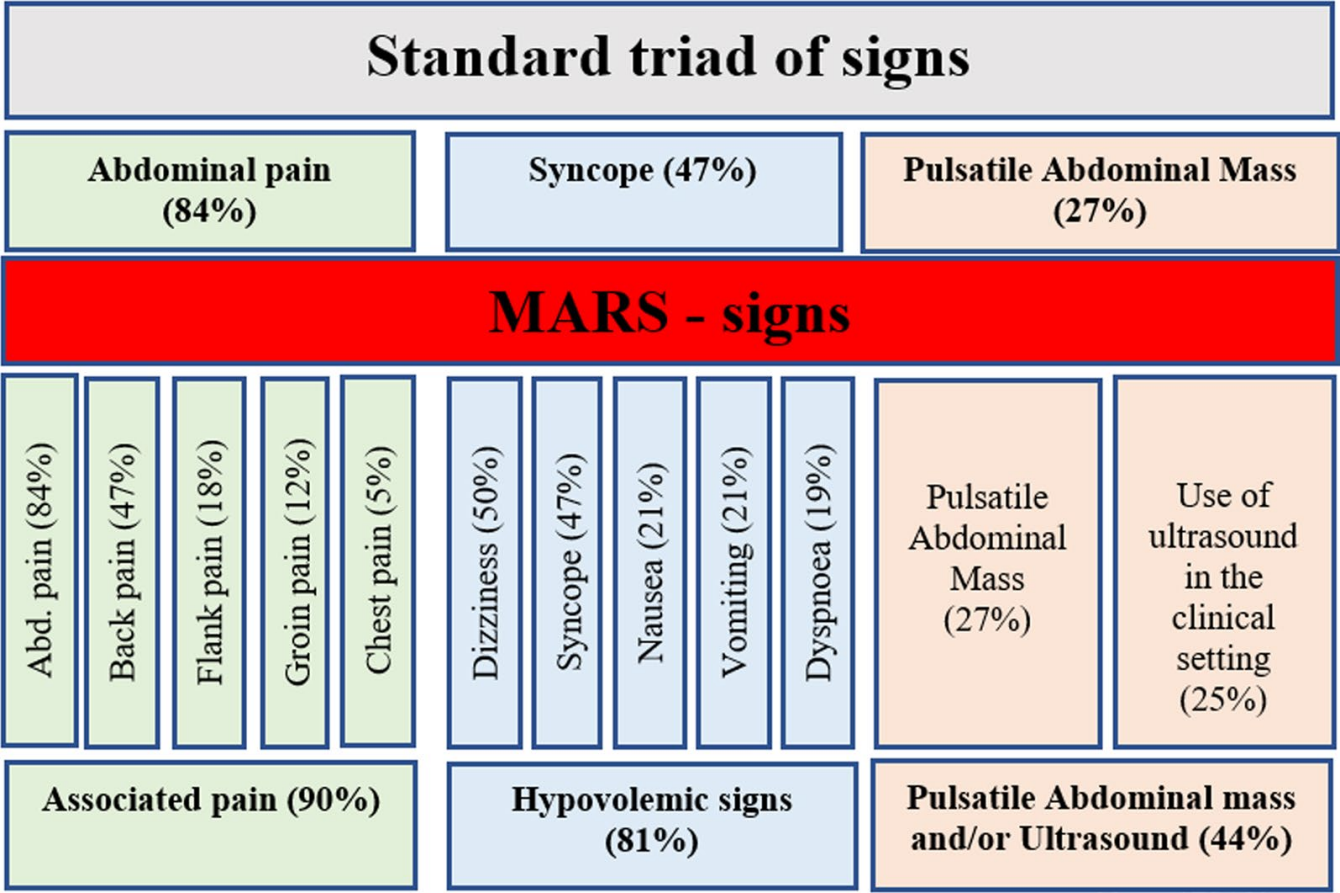
Summary and prevalence of symptoms for STS and MARS. Standard triad consist of abdominal pain, syncope and pulsatile abdominal mass. MARS includes all registries of any localization pain, expression of hypovolemic signs and addition of sonography or examination of pulsatile abdominal mass

**Fig. 3:**
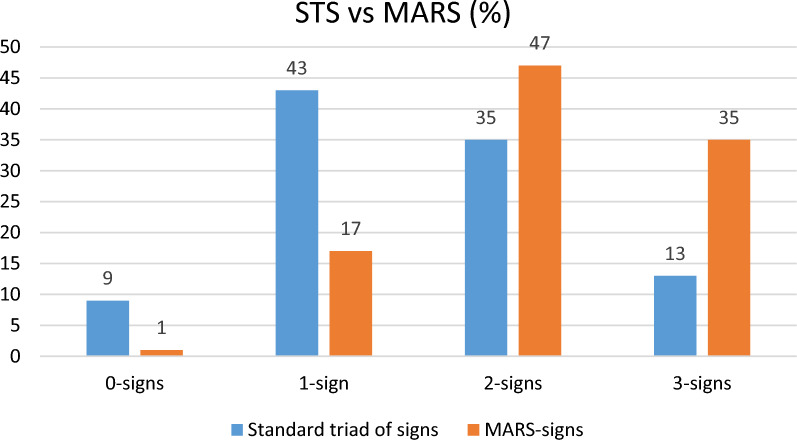
Comparison of the Standard triad of signs (STS) to Modified-Abdominal-Aortic aneurysm Rupture-Signs (MARS). *Comparison of 2 and 3 signs for STS vs MARS and 3 signs for STS vs MARS (*P* value < 0.001)

The current standard rAAA triad of signs include abdominal pain, syncope and pulsatile mass. The developed and proposed Modified-Abdominal Aortic Aneurysm-Rupture-Signs or MARS builds upon the current triad and includes the following:

All registries of pain (abdominal, back, flank, groin or chest) were included to “associated pain”. Similarly, syncope, dizziness, nausea, vomiting and dyspnea were sorted as surrogate symptoms indicating and related to “hypovolemic symptoms”. The clinical examination of the abdomen, which can indicate a pulsatile abdominal mass, can be enhanced by adjunct use of an aortic ultrasound, with transversal or longitudinal axis measuring the maximum infrarenal aortic diameter. This can be performed in the prehospital setting or at the emergency department (Figs. 2, 3). Most of the patients in this cohort were examined in the ER.

### Statistics

Continuous variables are reported as mean ± standard deviation or median and IQR (25–75 percentiles) depending on normal distribution tested with Shapiro–Wilk. Categorical data are presented using numbers and proportions. Statistical significance was tested for continuous data with Mann–Whitney U-test or t-test and for categorical data with Fisher’s test or Chi square depending on the number of patients. Two-tailed *P* value less than < 0.05 was considered significant. Statistical analysis was performed with IBM SPSS version 29.

## Results

### Patient characteristics

There were 216 patients with verified rAAA. Most of the patients were men (77%) with a mean age of 77 years (Table 1). The mean BMI was 26 and similar for both sexes. The mean age at rupture was higher in women, but the mean aortic diameters at rupture were smaller in women (Table 1). The prevalence of comorbidities did not significantly differ between the sexes, apart from chronic obstructive pulmonary disease being more prevalent in women (*P* 0.003) (Table 1). Open repair was chosen as the mode of surgery for 104 (48%) patients followed by EVAR for 89 patients. Six patients were primarily treated with EVAR but were converted to OR (Table 1).

**Table 1:** Characteristics for admitted patients with a verified ruptured abdominal aortic aneurysm

Characteristics	All 216 (100%)	Women 49 (23%)	Men 167 (77%)	*P* value
Age yrs. (median, IQR)	78 (13)	81 (13)	78 (12)	0.003
BMI (mean, ± SD)	26 ± (5.0)	27 ± (5.0)	27 ± (4.9)	0.81
Aortic diameter, mm (median, IQR)	75 (25)	75 (43)	75 ± (25)	0.52
Hypertension	152 (70)	39 (26)	113 (74)	0.11
Hyperlipidemia	91 (42)	17 (19)	74 (81)	0.23
Heart disease	128 (59)	27 (21)	101 (79)	0.5
Diabetes	38 (18)	8 (21)	30 (79)	0.79
COPD	39 (18)	16 (41)	23 (59)	0.003
Current smoker	54 (25)	14 (26)	40 (74)	0.09
Previous smoker	90 (42)	25 (28)	65 (72)	0.09
Treated	199 (92)	47 (95)	152 (91)	0.57
EVAR	89 (42)	18 (37)	71(43)	< 0.001

Data is presented as mean and standard deviations or median and interquartile range for continuous variables. Categorical data is presented as total numbers and proportions for each sex

Data presented for all patients with comparison between women and men separately

A 30-day mortality of 33% and 1-year mortality of 44% were recorded for the intervention groups without procedural or sex differences (Supplemental Table 1). No mortality predictive scoring systems were used in decision-making process for the operative choice.

### Symptoms in rAAA patients

The dominating registered signs was abdominal pain (84%), followed by dizziness (50%) and back pain (47%) (Table 2). Syncope was reported in 47% of the cases, and a pulsatile abdominal mass was noticed in 27% of the patients (Table 2).

**Table 2:** Reported symptoms detected in the electronic medical records (EMR) for patients with a verified ruptured abdominal aortic aneurysm, presented as the most common symptom to the least common

Symptoms	All 216 (100%)	Women 49 (23%)	Men 167 (77%)	*P* value
Abdominal pain	170 (84)	40 (82)	130 (78)	0.9
Dizziness	107 (50)	26 (53)	81 (49)	0.62
Back pain	102 (47)	27 (55)	75 (45)	0.43
Syncope	101 (47)	21 (43)	80 (48)	0.58
Pulsatile abdominal mass	58 (27)	14 (29)	44 (26)	0.53
Nausea	44 (21)	10 (20)	34 (20)	0.51
Vomiting	44 (21)	10 (20)	34 (20)	0.31
Dyspnoea	40 (19)	9 (18)	31 (19)	0.79
Flank pain	38 (18)	10 (20)	28 (17)	0.75
Groin pain	25 (12)	12 (24)	13 (8)	0.005
Chest pain	11 (5)	1 (2)	10 (6)	0.51

Data presented for all patients, women and men also presented and compared separately

Data is presented as numbers and proportions for each sex

Other commonly registered associated signs were nausea and vomiting, noted in a fifth of all patients.

### STS

The complete STS triad was found in 29/216 patients (13%). Around a third of all patients presented with two signs and 41% only had one of the triad signs (Table 3). Abdominal pain was the predominant symptom affecting 43% of women and 29% of the men as the only symptom, without other coexisting symptoms such as syncope and/or abdominal mass.

**Table 3:** Patients registered with 1, 2 or 3 of the standard triad of signs (STS) for ruptured abdominal aortic aneurysm (abdominal pain, syncope and/or abdominal mass)

Triad signs	All 216 (100%)	Women 49 (23%)	Men 167 (77%)	*P* value
1 Sign	88 (41%)	14 (29%)	74 (44%)	0.17
2 Signs	79 (37%)	23 (47%)	56 (34%)	
3 Signs	29 (13%)	6 (12%)	23 (14%)	

Data is presented as numbers and proportions for each sex

### MARS

When applying MARS, all signs of pain (abdominal, back, flank, groin, and chest pain) were grouped together (Fig. 2, Table 4). Similarly, signs associated with hypovolemia (syncope, dizziness, dyspnea, nausea, vomiting) were grouped together (Fig. 2, Table 4).

**Table 4:** Patients registered with Modified-Abdominal-Aortic aneurysm Rupture-Signs (MARS)

MARS-signs	All 216 (100%)	Women 49 (23%)	Men 167 (77%)	*P* value
Associated pain	194 (90)	46 (94)	148 (89)	0.42
Hypovolemic signs	176 (81)	41 (84)	135 (81)	0.65
Pulsatile abdominal mass or ultrasound	96 (44)	24 (49)	72 (43)	0.47
1 Sign	37 (17)	7 (14)	30 (18)	0.78
2 Signs	102 (47)	22 (45)	80 (48)	
3 Signs	75 (35)	20 (41)	55 (33)	

Thereafter the summarized signs are presented for all, women and men. The chosen collected symptoms classified as associated pain (abdominal, back, flank and chest pain), are grouped together. Symptoms associated to hypovolemia include, syncope, dizziness, dyspnoea, nausea or vomiting. A pulsatile abdominal mass at a clinical examination also includes an aortic sonography

Data is presented as numbers and proportions for each sex

More than one third of patients with rAAA fulfilled a complete MARS (35%), 47% of patients had 2 symptoms and 17% presented with one MARS. The comparison with fulfillment of STS vs MARS shows higher accuracy for MARS (3 signs 13 vs. 35%, *P* < 0.001, 2 or 3 signs 48 vs. 82%, *P* < 0.001) (Fig. 3).

### Sex differences

Groin pain was mostly reported in women and was also the only symptom that differed between the sexes (Table 2). A pulsatile abdominal mass was reported as often for men as for women (Table 2).

The distribution of STS varied slightly between the sexes, but these differences did not reach statistical significance (Table 3). With MARS applied to the cohort, no relevant sex differences were identified (Table 4).

## Discussion

The previously reported poor correlation between STS and a correct initial diagnosis of rAAA is confirmed in this cohort of patients and challenged when an expanded concept of closely associated signs are applied. This could assist in improving the emergent care trajectory of rAAA patients and ultimately influence survival rates.

Ruptured abdominal aortic aneurysm is one the most urgent surgical conditions in the emergency department, requiring immediate management [2]. Early detection and treatment could potentially save more lives. This cohort of patients with a verified rupture is similar to other cohorts of rAAA patients regarding the distribution of age, comorbidity, sex and mortality [3, 18, 23].

Our study and previous investigations indicate that only a minority of patients admitted to the ER with rAAA will present with the complete standard triad of signs [14–18].

In this cohort, approximately half of them registered common symptoms such as abdominal pain, dizziness, back pain and syncope. Despite the frequently reported symptoms, such as nausea and vomiting linked to hypovolemia, these indicators are disregarded.

The misdiagnosis or delay of a correct care trajectory for patients with fatal diagnoses such as rAAA has devastating consequences. There are, however, few reports on such associations in the literature. In one Swedish study from 2021, misdiagnosis was shown to contribute to a higher mortality rate as compared to a correct diagnosis in the emergency department (75 vs. 63%) [5].

By including the expanded MARS, one integrates various indicators of pain and common clinical interpretation of hypovolemic symptoms, alongside with the inclusion of aortic ultrasound. The development of this broad symptomatic interpretation was developed in response to a clinical apprehension of the too narrow and rigid STS scoring. This is clearly detected in the comparison of accuracy between STS and MARS for fulfillment of signs, 2 or 3 signs reached 82% with MARS vs 48% with STS.

Emergency physicians in ER are increasingly proficient with diagnostic tools like ultrasound, which supports the expansion of this tool in the emergency diagnosis of an rAAA*.* If an abdominal aortic aneurysm wider than 45 mm is diagnosed, this will support a more speedy care flow in the ER and decrease misdiagnosis of this challenging diagnostic group.

The mode of presentation, both hypotension and pain will differ dramatically based on the rupture site, which is a clinical experience among surgeons and has to some extent been reported by others previously [7, 13]. Whereas an intraperitoneal perforation causes exsanguination with abdominal pain and syncope a retroperitoneal rupture will cause a different clinical presentation. A retroperitoneal rupture is the most common rupture site with up to 80% of the cases [13, 14]. The classic retroperitoneal posterior tear can temporarily seal and result in a delayed hypovolemic presentation and show a different manifestation of pain. Due to the shape shifting nature of this disease and thus the widespread clinical presentation, as shown in this study, an expanded, yet clinically relevant diagnostic tool is needed to reduce the rates of misdiagnosis and mortality.

There are profound sex differences found in the rAAA patient group, the most important factor is the higher relative proportion of women in the ruptured groups compared to intact [3, 24, 25]. Further, the higher risk for women to die before admittance to hospital and the lower proportion possible to treat with EVAR has been reported [2, 3]. There is a lack of data on sex differences regarding symptoms, even if this cohort is relatively large, it is not possible to determine if there are any relevant clinical differences regarding presentation with rAAA for women and men.

## Limitations

There could be a difference in the distribution of the admitted rAAA population in Stockholm vs other urban areas worldwide, since there has been an ongoing screening program for 65-years old men since 2010. It is likely that the distribution of clinical signs is similar, regardless of the lower overall number of ruptures in the population due to the benefits of screening.

Although the cohort is relatively large, in-depth analysis of sex differences was not possible due to the usual expected smaller proportion of women.

## Conclusion

It is clear that by introducing a more generous understanding of the commonly applied STS, a higher chance of an earlier correct diagnosis could be anticipated. Further scientific evaluations of MARS in a clinical setting are warranted to explore the implementation of this scoring system in emergency care globally.

## Supplementary Information

Additional file 1.

## Data Availability

Yes, data is available upon request.
