# Supplementary material for: Initial signs in patients with ruptured abdominal aortic aneurysms: time for an expanded triad?
Source: Scand J Trauma Resusc Emerg Med. 2024 Sep 23;32:94. doi: 10.1186/s13049-024-01268-0 (PMC11421174; doi:10.1186/s13049-024-01268-0)
Supplement: Supplementary file 1 — Additional file 1. [file 13049_2024_1268_MOESM1_ESM.docx]

**Supplemental Table 1.** Comparison of treatment modality and mortality rates after 30 days and 1 year presented for EVAR and OR.

Patients treated with EVAR, and then converted to open repair are included into the open repair group.

|  | **All** | **Women** | **Men** |
| --- | --- | --- | --- |
| **30 d mort** | 72 (33%) | 19 (39%) | 53 (32%) |
| **1 y mort** | 96 (44%) | 23 (47%) | 73 (44%) |
| **30 d mort EVAR** | 38 (53%) | 7 (37%) | 29 (55%) |
| **30 d mort OR** | 30 (42%) | 11 (59%) | 19 (36%) |
| **1 y mort EVAR** | 44 (46%) | 8 (42%) | 36 (68%) |
| **1 y mort OR** | 42 (44%) | 14 (61%) | 30 (41%) |

*Data is presented as numbers and proportions for each gender.*
